# Systems validation: application to statistical programs

**DOI:** 10.1186/1471-2288-5-3

**Published:** 2005-01-13

**Authors:** Rickey E Carter

**Affiliations:** 1 Department of Biostatistics, Bioinformatics and Epidemiology, Medical University of South Carolina, Charleston, South Carolina, USA

## Abstract

**Background:**

In 2003, the United States Food and Drug Administration (FDA) released a guidance document on the scope of "Part 11" enforcement. In this guidance document, the FDA indicates an expectation of a risk-based approach to determining which systems should undergo validation. Since statistical programs manage and manipulate raw data, their implementation should be critically reviewed to determine whether or not they should undergo validation. However, the concepts of validation are not often discussed in biostatistics curriculum.

**Discussion:**

This paper summarizes a "Plan, Do, Say" approach to validation that can be incorporated into statistical training so that biostatisticians can understand and implement validation principles in their research.

**Summary:**

Validation is a process that requires dedicated attention. The process of validation can be easily understood in the context of the scientific method.

## Background

The United States Food and Drug Administration (FDA) has defined validation as "confirmation by examination and provision of objective evidence that computer system specifications conform to user needs and intended uses, and that all requirements can be consistently fulfilled" [1]. Validation is a process that begins with defining the requirements and ends with ensuring that the needs are being fulfilled consistently. Structured testing is only a part of the validation process. Interaction with researchers emphasized the notion that many people use validation and testing synonymously. To better illustrate the concepts of validation to these same researchers, the following mantra was developed:

Plan what you are going to do,

Do what you planned,

And

Say what you did.

As an introduction to the application of this mantra, consider the following. Clinical trial operations are detailed in numerous manuals and procedural documents with the protocol being the most notable. A clinical trial protocol serves as the master research plan. Great efforts will be employed, both by researchers and by external advisory boards, to ensure that the protocol is sound and is designed to support the clinical hypothesis. Conducting the protocol at clinical sites allows for the execution of the research methodology in a reproducible and orchestrated manner, and in the end, the final report or manuscript summarizes the work performed. If any one of these components is missing from the research plan, the scientific integrity of the research would be questioned or directly refuted. Associating systems validation to this well developed paradigm is at the heart of the "Plan, Do, Say" approach to validation. There are many good references available that detail the process of validation. Stokes' two books are excellent resources for investigators new to validation [2,3]. The FDA provides additional guidance to industry by publishing guidance documents that aid in the interpretation of federal regulations. National organizations such as the Drug Information Association routinely host short sessions addressing validation. Still, for many practicing biostatisticians or other researchers, the concepts of validation may not be clearly understood. Therefore, the intent of this manuscript is to offer guidance on what is a validated computerized system to these individuals and provide a common framework that will enable effective communication.

When revisiting the FDA definition of validation in the context of the scientific method, it becomes clear that "confirmation by examination and provision of objective evidence that the computer system specifications conform to user needs and intended uses, and that all requirements can be consistently fulfilled" is essentially applying the scientific method to the life cycle of computerized systems. The definition implies that a validated system is one in which processes are specific and clearly defined, reproducible and consistently applied, and result in a measurable and observable level of quality in the delivered product. Validation of applicable systems is viewed as the best way to ensure that research objectives can be consistently met.

All clinical trials need to collect and deliver results in an efficient and accurate manner while ensuring the integrity of the data. The CONSORT Statement provides guidance to researchers on the format and content of clinical trial reporting [4]. Computerized systems validation is integral to the fulfillment of the CONSORT Statement since a validation process for statistical programs will make the analysis understandable and reproducible by external reviewers. It is the responsibility of the computer system owner to ensure that the system is properly validated. For statistical reporting of biomedical data, the computer system owner is the senior trial biostatistician in consultation with sponsor and principal investigators.

In addition, validation should be considered as a part of the complete life cycle of a computerized system [2]. This life cycle includes the stages of planning, specifying, programming or purchasing, testing, documenting, operating, monitoring and modifying as necessary. The primary focus of regulatory inspection for computerized system validation is to obtain documented evidence to assure that any system is currently operating properly.

## Discussion

Just as the "Plan, Do, Say" mantra illustrates, there are three main validation deliverables [5]. First, a validation plan must be developed. The validation plan should include the general scope of the system including high-level design features, the personnel responsible for the validation, a timeline, and a summary of other supporting documentation that need to be addressed in order to use the system. The whole deliverable must be approved by management prior to its deployment [5]. Second, there should be documentation that the system has been designed as envisioned in the validation plan [5]. There should be at a minimum complete system specifications / requirements, a traceability matrix and test scripts included in this set of documentation. A test script should be written to ensure that the system requirements function as desired by measuring whether the system produces the expected result, and a traceability matrix cross references the system requirements to individual test script items. Finally, a report should be included with the validation deliverables. The whole deliverable must be approved by management and indicate that the system functions as required [5]. A report also is a place to discuss how deviations to the plan were addressed.

The scope and magnitude of the delieverable will vary from computerized system to computerized system, and it is the responsibility of the management team to decide upon the level. The following section provides an outline to serve as an aid to assembling the validation deliverables for statistical programs.

### Statistical program checklist

1. Planning activities

(a) Develop a validation plan

State how the validation process will be conducted, including personnel roles, test parameters, and decision points on what constitutes acceptable test results. Management prior to the development of the detailed specifications should approve the validation plan. The plan may be amended with time.

(b) Utilize standard operating procedures (SOPs)

SOPs should be available to formalize the procedures used in the validation process as well as establish statistical programming standards. Incorporation of standards will aid in producing reproducible deliverables. The following is a partial list of applicable SOPs:

i. Validation of statistical programs

ii. Statistical programming standards

iii. Statistical program archival: Outlines the necessary steps to archive the analysis program, data sets, and, if necessary, computer hardware so that the results may be reconfirmed at a future date.

(c) Document training on SOPs

Written SOPs are not useful unless they are incorporated into practice. In order to do this, applicable individuals need to be orientated to the procedures. This orientation session should be documented for regulatory inspection.

(d) Develop detailed specifications

i. Data management plan

A. Annotated database structure

B. List of coding conventions for data

C. List of procedures used to process the data

D. Merging criteria (database keys)

E. System environment: analysis package (with version), system hardware, input data structure, output data structure, long-term storage environment

ii. Analysis objectives

The analysis objectives may vary according to the application; however, for the primary clinical trial report, the protocol or a statistical analysis plan may be sufficient to detail the requirements of the analysis.

(e) Develop a test plan and/or test script

i. Mock tables

ii. Expected results of tests

iii. Programming standards

iv. Testing procedures

2. Execution of the plan

(a) Retain raw test results

Record individual pass/fail for each step outlined in the test script. A pass occurs when the observed result matches the expected result.

(b) Note variances and deviations to the test plan

(c) Document location, time and individuals involved in the testing process

3. Summary report

(a) Summarize validation process

(b) Summarize the variances and deviations

(c) Summarize test results and provide interpretation when necessary

(d) Approve by management

### Application to statistical programs

The definition of a computerized system encompasses both the hardware and software. For analytical applications, the key components of the system also will include the individual programs written to perform the analysis. The use of validated macros aides in reducing the burden introduced by the validation process for an individual system. For example, suppose for the clinical reporting of a trial's results, a table is desired that reports the mean and standard deviation or count and percentage for all putative covariates. A SAS macro [6] could be written to compile and export the data from SAS to a word-processing compatible format. This macro would be a candidate for validation since it manipulates raw data, performs calculations, and modifies output from standard SAS output. However, once this macro has been developed, a significant savings in the time required to produce (and verify) publication-ready tables could be possible. The remainder of the discussion highlights some of the key steps in validating this macro.

The validation of the macro begins in the planning stage. For the macro to "conform to user needs", the needs need to be clearly identified. The following may describe its user requirements:

1. Export a word-processing compatible table consisting of three columns;

2. The table columns must be appropriately labeled;

3. Column 1 must be the labeled "Characteristic";

4. Column 2 must be the labeled "Control Group";

5. Column 3 must be the labeled "Intervention Group";

6. For categorical covariates, calculate the count and percentage for each level of the variable;

7. For levels of a categorical variable, the table should indent the formatted description; and

8. For interval-scaled continuous covariates, calculate the mean and standard deviation.

This set of requirements would then be discussed from a technical perspective. Issues such has how to best calculate the summary statistics could be a topic of discussion. The use of PROC TABULATE and the Output Delivery System (ODS) [6] could be a likely candidate for the summary. Next, discussion pertaining to the specification of covariates and the concatenation of the results is needed. In the process, potential macro parameters would need to be identified. All key decisions are incorporated into the validation plan. This plan is reviewed by the computer system owner and the validation system sponsor.

Once this plan has been approved, a revision log should be kept to ensure traceability of changes that may occur during development.

Once the macro has been developed, the validation system sponsor should work either independently or in conjunction with appropriate staff to review the programming and develop test cases. The test cases are assembled into a test that specifies the system input and expected output. To ensure all user requirements are addressed, a traceability matrix can be utilized. A simple traceability matrix for this validation exercise would list each of the requirements and cross reference the applicable test.

The test script is then implemented under the direction of the validation system sponsor. Documentation of the event should be recorded so that it can be compiled into the validation report. Any discrepancies between the observed and expected results need to be addressed, and, if applicable, documentation on the corrective actions employed to resolve the issue(s) needs to be compiled into the validation deliverables. A revision log of programming changes is desirable; however, in practice, this may prove difficult to document completely. Once the testing has been completed, the computer system owner and validation system sponsor can determine what additional steps are required prior to release. For SAS macros, it is critical to document the parameters, default values of the parameters and system dependencies, then, perhaps include illustrative examples of the macro's use. Finally, a written statement by the computer system owner and the validation system sponsor should be issued stating that the macro has been validated and has been deemed acceptable for use.

The above example illustrates a full-scale implementation of a validation process. However, it is acknowledged that individuals and organizations must decide the level of validation that is required. In many cases, more traditional approaches of program verification (code review, double programming, etc.) may be sufficient. The importance of the "Plan, Do, Say" mantra is that procedures used for validation are specified and operated on.

## Summary

The "Plan, Do, Say" approach presented addresses the key deliverables an auditor expects to see in validated systems. By organizing validation activities into a plan, an action or a summary, one begins to gain efficiencies through proper planning. However, individual researchers and support organizations need to evaluate their particular needs for validation and develop a set of procedures to support their needs. The intent of this article is not to provide an ironclad approach to validation that will ultimately meet all needs. However, this design for validation has been conveyed successfully to a variety of audiences. The analogy of the scientific method helps break the technology and nomenclature barrier associated with a more computer science-driven approach to systems validation by associating the importance of validation to the process of designing a research plan.

## Competing interests

The author(s) declare that they have no competing interests.

## Pre-publication history

The pre-publication history for this paper can be accessed here:


